# Invasive *Aspergillus niger* Is the Sole Etiological Agent for CSOM : A Clinical Case from Nepal

**DOI:** 10.1155/2021/5556679

**Published:** 2021-09-27

**Authors:** Ajay Kumar Chaurasiya, Rabindra Bhakta Pradhananga, Niranjan Prasad Sah, Basista Prasad Rijal, Bharat Mani Pokhrel, Santosh Dulal

**Affiliations:** ^1^Department of Microbiology, Tribhuvan University and Teaching Hospital (TUTH), Institute of Medicine, Kathmandu, Nepal; ^2^Department of Microbiology, Patan Academy of Health Sciences (PAHS), Lalitpur, Nepal; ^3^Department of Otorhinolaryngology-Head and Neck Surgery (ENT-HNS), Tribhuvan University and Teaching Hospital (TUTH), Institute of Medicine, Kathmandu, Nepal; ^4^Medical Microbiology Program, Nobel College, Pokhara University, Sinamangal, Kathmandu, Nepal

## Abstract

*Aspergillus* causing chronic suppurative otitis media (CSOM) is rare in immunocompetent people; however, it can occur as a significant opportunistic pathogen in immunocompromised patients. Here, in our study, a 53-year-old diabetic patient having a history of CSOM visited the Department of Otorhinolaryngology-Head and Neck Surgery (ENT-HNS), Tribhuvan University and Teaching Hospital (TUTH), Nepal, in March 2016. Although he was on medication with an antibacterial ear drop from the last 10 days, his right ear was presented with otorrhea, pruritus, otalgia, aural fullness, hearing impairment, and tinnitus from the last 3-4 months. Preliminarily, otoscopy of the right ear revealed the presence of fungal mass. For further diagnosis, ear discharge was aseptically collected and sent to the laboratory to confirm the etiological agents. Findings of laboratory analysis indicated that Gram staining of aural discharge displayed pus cells with fungal spores but did not exhibit bacteria. Furthermore, potassium hydroxide (KOH) mount revealed the presence of fungal spores and septate hyphae with the characteristic of dichotomous branching. Culture in four different bacterial media (chocolate agar, blood agar, MacConkey agar, and Robertson's cooked meat medium) has unveiled no bacterial growth. However, fungal growth was observed in both bacterial and fungal media. Thereafter, the fungal colony was investigated via a lactophenol cotton blue (LPCB) tease mount which displayed the structure of *Aspergillus*. *Aspergillus niger* was microbially conformed by specifically characterizing the specific phenotypic biseriate structure of phialides and the black-coloured conidia. For medication, the patient was treated with Candid Ear Drop with clotrimazole (1% w/v) plus lidocaine (2% w/v) for 4 weeks which successfully improved his condition.

## 1. Introduction

Chronic suppurative otitis media (CSOM) is a chronic inflammatory middle ear cleft condition that is aggravated by partial or complete loss of the tympanic membrane and ossicles and often induces permanent sequelae that manifest as deafness and persistent ear discharge [1]. Globally, the burden of CSOM illness accounts for 28000 deaths; 65–330 million with draining ear and 39–200 million people are suffering from significant hearing impairment [2]. In Nepal, it is a major public health burden with a high prevalence rate [3]. If the prevalence of CSOM is greater than 3, it is accounted as a high-risk priority disease in the region. Nepal falls in the high endemic of CSOM with a prevalence of 3.5% [3–5]. We are reporting a case of a CSOM patient who was initially treated with topical antibiotics, whereas the laboratory diagnosis was secured on the evaluation for fungal pathogenesis and revealed invasive *Aspergillus niger* as the sole etiological agent. We, therefore, recommend a strong indicator of clinical suspicion supported by the standard and relevant microbiological analysis to avoid its misdiagnosis for an effective treatment option.

## 2. Case Presentation

A 53-year-old man who has a history of CSOM in the right ear was suffering for more than 3 months. He had visited various healthcare facilities and hospitals. He was on an antibacterial drug from the last 10 days but was not benefited. Finally, he visited the department of ENT and Head and Neck Surgery OPD of a tertiary-care hospital and underwent clinical investigation and treatment in March 2016.

## 3. Investigation and Outcomes

During his visit to the Department of Otorhinolaryngology-Head and Neck Surgery (ENT-HNS), Tribhuvan University and Teaching Hospital (TUTH), a medical team observed his ears with an otoscope and immediately spotted the ear infection with fungus, i.e., otomycosis. Furthermore, the doctor performed microscopic suction clearance of fungal mass. Subsequently, the use of topical antibiotic was discontinued.

For clinical investigation, we aseptically collected four swab samples from the right ear discharge and conducted further downstream assays, primarily microbiological investigations, Gram stain, KOH mount, and bacterial and fungal culture of aural discharge. The patient was advised for follow-up after a week for further treatment.

The first aural swab was processed for the preparation of Gram staining and potassium hydroxide (KOH) mount preparation. The second swab was inoculated into Robertson's cooked meat (RCM) medium, while the third was aliquoted into the nutrient broth (NB) and then the aliquots were inoculated into chocolate agar (CHOC), 5% sheep blood agar (BAP), and MacConkey medium (MAC), respectively. The fourth swab was utilized for the culture in Sabouraud dextrose agar (SDA), and clonal selection and subculture were conducted in specific media, i.e., malt yeast agar (MYA) and corn meal agar (CMA). All the clinical investigation procedures have been performed in accordance with the guidelines of the Clinical Microbiology Procedures Handbook, Volume 2 by Lynne S. Garcia, and Textbook of Medical Mycology, the Third Edition by Jagdish Chander. All the experiments were performed in triplicate utilizing the relevant positive and negative controls.

**Figure 1:**
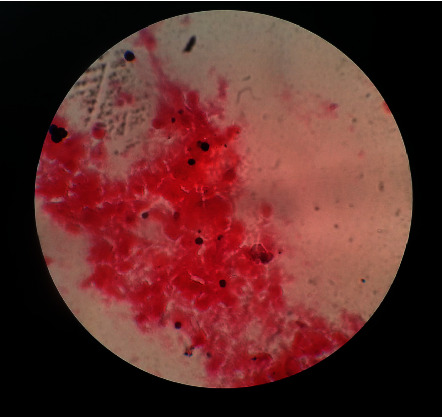
Gram staining revealing plenty of pus cells with fungal spores (Conidia) but lacking bacteria, when observed under a 100X Olympus CX21 compound microscope.

The Gram staining of the aural discharge revealed the presence of plenty of pus cells with fungal spores (conidia); however, no bacteria were detected (Figure 1).

**Figure 2:**
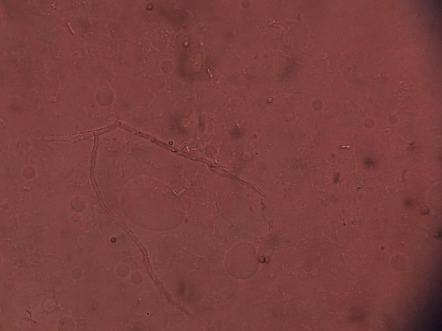
KOH mount revealing the septate fungal hyphae with the characteristic dichotomous branching, when observed under a 40X Olympus CX21 compound microscope.

**Figure 3:**
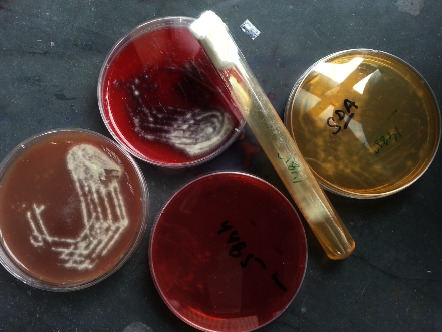
Aliquots from the culture sample grown on CHOC, BAP, MAC, and SDA media indicate the growth of *Aspergillus* spp.

The KOH mount exhibited the septate fungal hyphae with dichotomous branching (a typical feature of *Aspergillus* spp.) (Figure 2). For further microbial characterization and investigation, the aliquot of the culture was incubated for 18–24 hours in all three media plates, i.e., chocolate agar, blood agar, and MacConkey agar. The results indicated that there was no bacterial growth; however, fungal growth was observed in all three media plates (Figure 3).

To rule out bacterial a growth, Gram stain was performed with the colonies/growth from all three media plates. For the precise phenotypic characterization of the fungal growth, LPCB preparation was assessed and observed.

After 48 hours of incubation in RCM, the growth was further subcultivated into blood agar with disks of metronidazole plus gentamicin and cultured anaerobically for 48 hours. *Pseudomonas aeruginosa* (previously isolated in the department) was used as a control strain for aerobic culture and *Clostridium sporogenes* (previously isolated in the department) was used as a control for anaerobic culture in this study.

In the blood agar medium, no bacterial growth was observed; however, the light growth of the fungus was spotted which was conformed through Gram staining and LPCB mount preparation.

**Figure 4:**
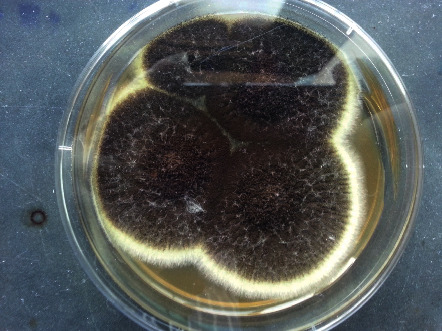
*Aspergillus* spp. observed as dark brown/black colonies grown on the SDA medium.

**Figure 5:**
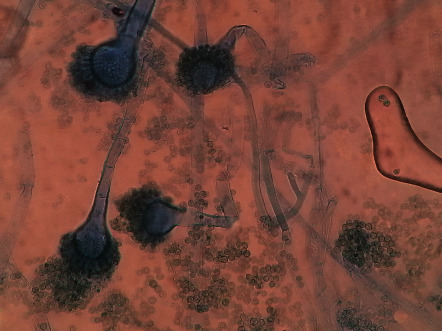
Confirmation of *Aspergillus niger* with the specific phenotypic characterization of conidiophores, vesicles, metulae, biseriate phialides, and conidia in the LPCB mount, observed under a 100X Olympus CX21 compound microscope.

After 48 hours of incubation in an SDA medium, the fungal growth (Figure 4) was further inoculated into a corn meal agar (CMA) and cultured for 72 hours. The fungal colonies from SDA and CMA media were proceeded into the lactophenol cotton blue (LPCB) tease mount. The microscopy results from the LPCB mount revealed the presence of multiple conidia, conidiophores, vesicles, phialides, and metulae resembling *Aspergillus* spp. (Figure 5). Furthermore, we have also conducted the clonal selection and utilized Malt yeast agar (MYA) and CMA-specific culture media for the growth of *Aspergillus* spp. and successfully isolated and confirmed *Aspergillus niger*. From all microbial observations and analysis, the finding of the vital characterization of the specific biseriate structure of phialides and the black-coloured conidia phenotypically confirmed *Aspergillus niger* as the sole etiological agent for this CSOM.

## 4. Discussion

Otomycosis is a chronic recurring mycosis of the outer ear canal. The incidence rate of otomycosis is high in tropical regions, and the species of *Aspergillus*are considered the predominant etiological factors which account for 48.9% of the entire otomycosis manifestations [6].

CSOM is one of the chronic types of otitis. It is ubiquitously caused by specific bacteria and fungi. Etiological agents of CSOM of significant aerobic bacterial origins are *Pseudomonas aeruginosa*, *Staphylococcus aureus*, *Proteus mirabilis*, *Klebsiella pneumoniae*, and *Escherichia coli*, while anaerobic bacterial origins are *Clostridium* spp., *Peptostreptococcus* spp., *Fusobacterium* spp., and *Veillonella* spp. Major fungal species involved in CSOM are *Aspergillus* spp., *Candida* spp., *Rhizopus s*pp., and *Penicillium* spp. [7, 8]. Prolonged and irrational use of topical antimicrobial (antibiotics or antibiotics-steroids) ear drops could shift and suppress the commensal and emerge opportunistic bacterial flora [9].

CSOM is a common health burden in low- and middle-income countries where socioeconomic status, overall health, hygiene and sanitation conditions, nutritional status, and accessibility to healthcare services are severely compromised. In addition, frequent upper respiratory tract infections, irrational antibiotic treatment, and nasal diseases are considered as the vital predisposing factors to CSOM [10, 11]. Without a precise etiological diagnosis of CSOM, the irrational use of antibiotics leads to the emergence of multidrug-resistant microbial strains which gives rise to severe complications that is an ultimate challenge currently faced by low- and middle-income countries. Each year, CSOM accounts for thousands of deaths and directly impacts millions of people who suffer from significant hearing impairment.

*Aspergillus* species are the most predominant fungal etiology in CSOM. *Aspergillus* conidia disperse in airborne dust, colonize, and grow rapidly in the ear canal which is supported by nutrients present in cerumen and the slightly acidic environmental condition. Hundreds of *Aspergillus* conidia can survive within the air; therefore, even if it is inhaled, no disease can be progressed to the immune-competent individuals. However, the immunocompromised hosts with diabetes, steroid administration, HIV infection, chemotherapy, and malignancy are susceptible to CSOM with fungal infection [12]. Immunosuppression health condition increases the risk of dissemination of the opportunistic pathogens *Aspergillus* spp. into various body organs either via circulation or by direct tissue invasion [13]. *Aspergillus* species ubiquitously occur in the diverse environment and their conidia mostly disperse in the air. If anyone is exposed to these conidia, the risk of otomycosis/CSOM progression is minimal for immune-competent individuals [14]. *Aspergillus niger* is one of the significant and predominant invasive fungal pathogens responsible for the infection of recurrent drainage of CSOM.

The proper clinical diagnosis of CSOM is carried out by the standard bacteriological and mycological assessments. Microbial phenotypic studies specifically focusing on fungal culture, wet mount, and staining are able to distinguish the specific etiological agent of CSOM in resource-constraint laboratory settings. Therefore, the clinical investigation and diagnosis of the etiological agent of CSOM can be achieved by the observation/demonstration of microbial evidence by the variety of standard methods, primarily Gram staining, KOH mount preparation, and bacterial and fungal culture in selective and differential media.

**Figure 6:**
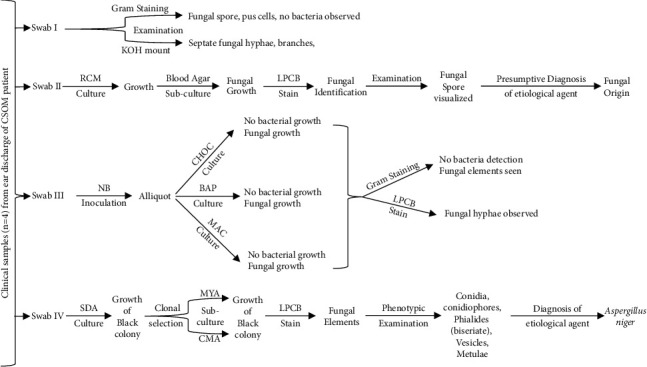
Flowchart showing overall laboratory investigation assessments and result outcomes in this study.

In this case study, we have clinically investigated the aural discharge from the right ear of a 53-year-old man suffering from CSOM. The patient probably got infected being immunocompromised due to his diabetic condition. We have systematically investigated this case study with the standardized microbial assessments which is shown in the flowchart for this study (Figure 6). The findings revealed no bacterial growth was able to be cultured from the collected samples of the patient; however, the fungus was isolated and identified.

Clonal selection and the specific culture in MYA and CMA generated black colonies of *Aspergillus* spp. which was further phenotypically investigated. For further characterization of *Aspergillus* spp., the revelation of the specific phenotypic characterization of conidiophores, vesicles, metulae, biseriate phialides, and conidia in microscopy phenotypically confirmed *Aspergillus niger* as the sole etiological agent of this case of CSOM.

The current recommended treatment for otomycosis due to *Aspergillus* spp. is clotrimazole (1% w/v) ear drops, and they are given for 3-4 weeks. It is considered as the effective antifungal therapeutic option as it stops fungal growth in the ear blocking them from the formation of their protective covering [15, 16].

For the therapy of this clinical case, the patient was also treated with the recommended medication of Candid Ear Drop with lidocaine (2% w/v) plus clotrimazole (1% w/v) for 4 weeks. When we followed up after 4 weeks of treatment and made the patient undergo overall ear check-up investigations, his ear and health conditions were improved remarkably. For further clinical investigation, swab samples were also aseptically collected from both ears and microbial assessments were performed; however, no microbial growth was isolated and detected.

## 5. Conclusions

CSOM caused by both bacterial and fungal origins in laboratory diagnosis has frequently been reported; however, CSOM associated with the clinically relevant fungal flora alone is a unique case.

In this clinical case report, we have clinically and microbiologically investigated the aural discharge from a CSOM patient, isolated and phenotypically distinguished invasive *Aspergillus niger* as the sole etiological pathogen associated with the CSOM presented.

To sum up, irrational and prolonged use of antimicrobials ear drops may lead to the emergence of the microbial superinfection-CSOM with opportunistic virulent pathogens. Therefore, a definite and prompt investigation for fungal etiology with the standardized laboratory assessments is recommended for each CSOM case which subsequently guides for the proper and timely management and treatment of CSOM and prevents CSOM-associated severe complications.

## Data Availability

No data were used to support this study.

## Abbreviations

BAP: 5% sheep blood agar plate
CMA: Corn meal agar
CHOC: Chocolate agar
CSOM: Chronic suppurative otitis media
ENT-HNS: Otorhinolaryngology-head and neck surgery
HIV: Human immunodeficiency virus
KOH: Potassium hydroxide
LPCB: Lactophenol cotton blue
MAC: MacConkey agar
MYA: Malt yeast agar
NB: Nutrient broth
OPD: Outpatient department
RCM: Robertson's cooked meat
SDA: Sabouraud dextrose agar
TUTH: Tribhuvan University Teaching Hospital.
